# Supplementary material for: All-metallic magnetic Purcell enhancement in a thermally stable room-temperature maser
Source: Nat Commun. 2025 Dec 10;16:11214. doi: 10.1038/s41467-025-66016-z (PMC12715216; doi:10.1038/s41467-025-66016-z)
Supplement: Supplementary file 1 — Supplementary Information [file 41467_2025_66016_MOESM1_ESM.pdf]

Supplementary materials: All-metallic magnetic Purcell enhancement in a thermally stable room-temperature maser

Rongrong Xiang, Philippe Bugnon, Maliheh Khatibi Moghaddam, and Romain Fleury*

Laboratory of Wave Engineering, École Polytechnique Fédérale de Lausanne, Lausanne, Switzerland

(Dated: October 27, 2025)

Contents

SI. Supplementary Figures 1

SII. Supplementary Discussion 6

A. Statistical Evaluation of the Mode Uniformity in the Resonator Bore Region 6

B. Thermal Stability for Continuous-Wave Maser Operation 6

SI. Supplementary Figures

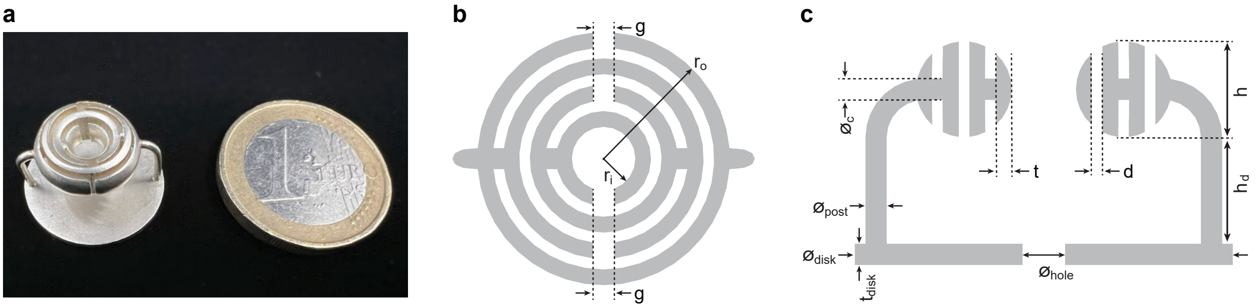

Figure S1: Geometry of the toroidal SRR cluster resonating at approximately 1.45 GHz ($\lambda_m \sim 207$ mm). a, A photograph of the all-metallic SRR cluster that is manufactured in silver. The detailed dimensions are given in the top view **b** and cross-section view **c** of the cluster. The optimized SRR cluster has a donut-like shape with inner radius $r_i = 1.52$ mm, outer radius $r_o = 6.02$ mm and height h = 4.5 mm, ensuring the optimal magnetic field distribution. The cluster consists of four concentric split rings with a split gap g = 1 mm. Each ring has a maximum thickness of t = 0.75 mm and is spaced from the adjacent rings by d = 0.5 mm. To facilitate monolithic fabrication and avoid post-fabrication alignment, the adjacent rings are connected by a small rod ($\phi_c = 1$ mm) and the toroidal cluster is affixed onto a disk ($\phi_{disk} = 17.8$ mm, $t_{disk} = 1$ mm) with a distance $h_d = 5$ mm using two thin posts ($\phi_{post} = 1$ mm). In addition, the bottom disk has a central hole ($\phi_{hole} = 2$ mm) for inserting the optical fiber.

* Corresponding author. E-mail:romain.fleury@epfl.ch

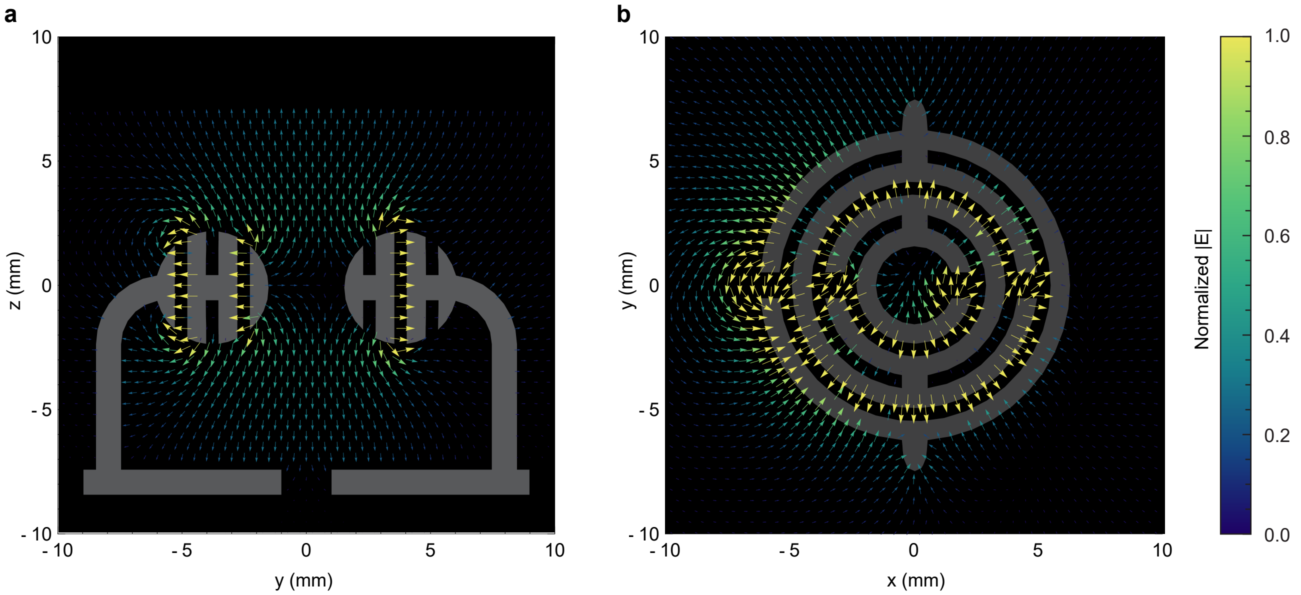

Figure S2: Electric field distribution within the cross section of the toroidal SRR cluster in the yz-plane (a) and xy-plane (b).

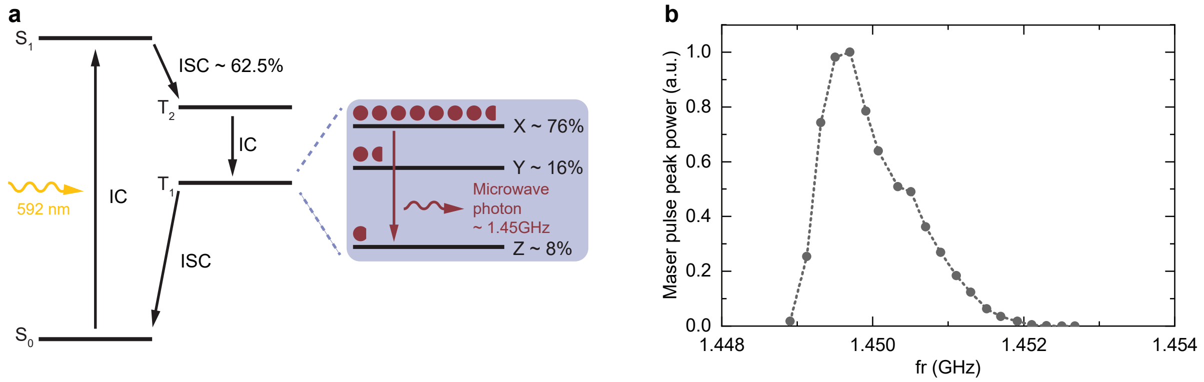

Figure S3: Masing in pentacene molecules hosted in a p-terphenyl crystal. a, The simplified Jablonski diagram describes the masing process in pentacene molecules. Optical excitation at 592 nm pumps pentacene molecules from the singlet ground state S_0 into the singlet excited states S_1 via internal conversion (IC). Approximately 62.5% of the excited molecules decay into the second triplet state T_2 via intersystem crossing (ISC) with intrinsic spin-polarized population, which then transitions into sublevels X, Y and Z in the first triplet state T_1 meanwhile preserving the inverted spin ratio (X:Y:Z = 0.76:0.16:0.08). When placed in a cavity resonating at around 1.45 GHz, maser transitions occur between X and Z by depleting the population inversion. **b,** Measured gain linewidth of pentacene molecules. The strongest maser response occurs at about 1.4496 GHz, with a full width of half maximum (FWHM) of approximately 1 MHz.

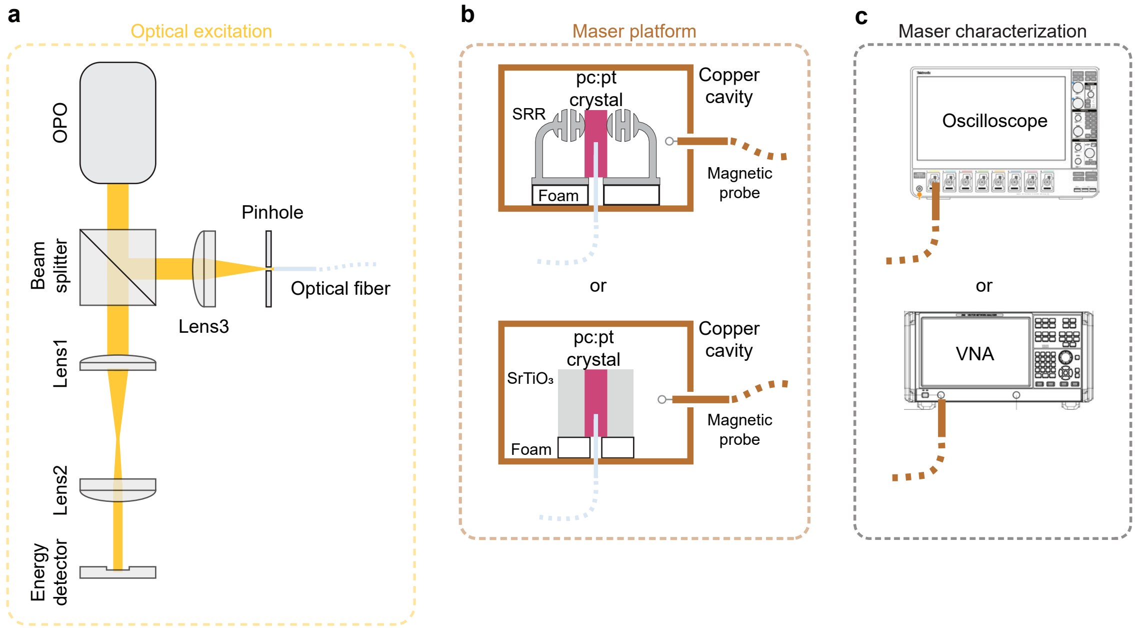

Figure S4: Complete experimental configuration. a, Optical excitation. The output beam from an optical parametric oscillator (OPO) is divided using a beam splitter (T:R $\sim$ 15%:85%): the transmitted beam is shrunk using a telescope and measured by a energy sensor (Gentec-EO, Pyroelectric detector QE12LP-S-MB-QED-INE-D0); the reflected beam is coupled into a multi-mode optical fiber (core diameter 1mm) using a plano-convex lens. To filter the spatial profile of the focused beam and protect the optical fiber, a precise pinhole (hole diameter 1 mm) is placed in front of the optical fiber. The other end of the optical fiber enters the pentacene:p-terphenyl (pc:pt) crystal for efficient optical pumping. **b,** To maintain consistent experimental conditions, the clustered-SRR- and $SrTiO_3$-based masers share the same optical excitation and characterization setup. A copper cavity, with a radius of 18 mm and an adjustable height from 15 mm to 40 mm, is employed to house the resonators. Details on the clustered-SRR-based maser have been presented in the main text and Fig.S1. In the $SrTiO_3$-based maser, a hollow cylindrical $SrTiO_3$ resonator (SurfaceNet GmbH), with and inner radius of 1.5 mm, an outer radius of 5.35 mm and a height of 11 mm, is placed on a 6-mm high spacer. This spacer is made of an air-like low-loss Polyethylene foam (Eccostock PP) and it has a central hole allowing penetration of the optical fiber. The fundamental $TE_{01\delta}$ mode of the $SrTiO_3$ resonator has an unloaded Q factor around 9000 and a magnetic mode volume $V_m \sim 0.25$ cm^3 at approximately 1.45 GHz. When weakly coupled to the magnetic probe, the loaded Q factor of the $SrTiO_3$ resonator is around 7200. **c,** Characterization of the maser platforms. Prior to and after the maser experiment, the magnetic probe is connected to the a vector network analyzer (VNA) to measure the S_{11} parameter of the maser platforms without optical excitation. During the maser experiment, maser signals captured by the magnetic probe are recorded using an oscilloscope, which is electrically triggered by the pump laser.

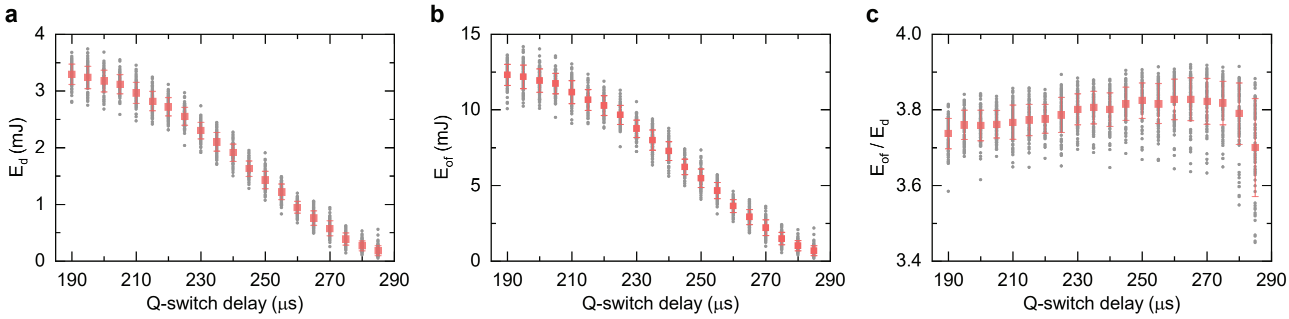

Figure S5: Fluctuations in the output beam from the OPO at different Q-switch delay. Prior to each maser experiment, the output energy from the optical fiber (E_{of}) **a** and energy transmitted by the beam splitter (E_d) **b** are measured to calibrate the energy ratio ($\zeta = E_{of}/E_d$) **c**. By increasing the Q-switch delay time, the output energy from the OPO can be decreased. For each Q-switch delay time, we record 100 optical pulses at a repetition rate of 1 Hz. However, due to inconsistencies in the output beam profile from the OPO, the amount of energy coupled into the optical fiber varies in each pulse, introducing fluctuations in the value of ζ. During the maser experiment, the actual excitation energy E_{of} delivered into the pc:pt crystal is estimated from the laser energy E_d detected correspondingly using the previously calibrated value of ζ. Consequently, fluctuations in ζ lead to uncertainties on the actual pumping energy.

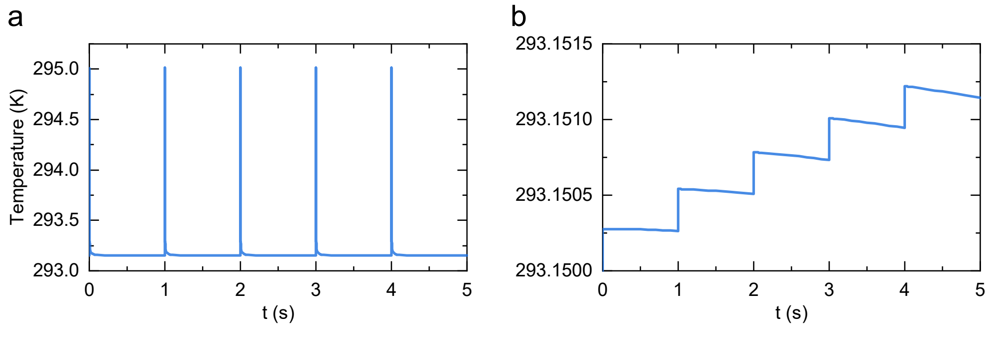

Figure S6: Maximum (a) and average (b) temperature variations of the $SrTiO_3$ resonator under optical excitation (COMSOL simulation) over a five-second interval. The pump laser operates at a repetition rate of 1 Hz, with a pulse duration of 5 ns and a pulse energy of 3 mJ. The laser is active during the first 5 ns of each second, heating the $SrTiO_3$ resonator.

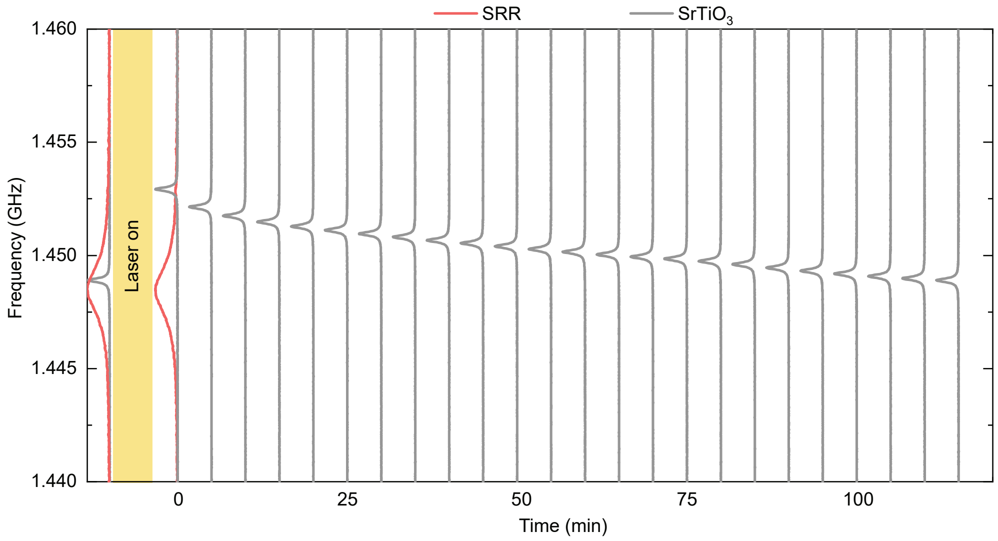

Figure S7: S_{11} parameters measured in the clustered-SRR- and $SrTiO_3$-based masers (red and grey curves, respectively) prior to and following optical excitation. The leftmost S_{11} curves were measured using the VNA before the pump laser was switched on. After the masing experiment (on the right side of the yellow zone), the S_{11} parameters of both masers were measured immediately after switching off the pump laser(denoted as 0 min). Since the spectral response of the clustered-SRR-based maser remained unchanged throughout the masing process, only the S_{11} parameter of the $SrTiO_3$-based maser was consecutively measured every 5 minutes for a duration of 115 minutes until it returned to its initial value.

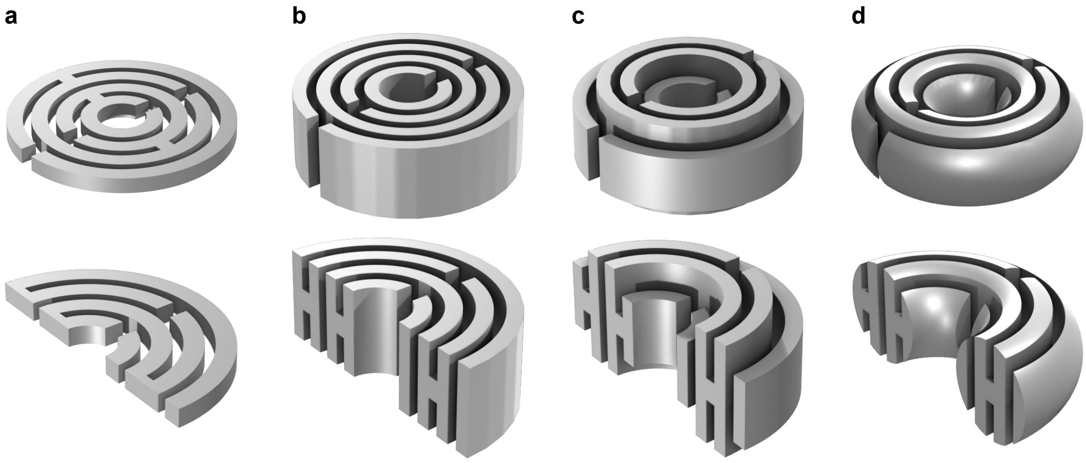

Figure S8: Progressive evolution of the resonator design. a, Planar SRR cluster: The number of concentric rings is increased to shift the resonant frequency near 2 GHz. **b,** Cylindrical SRR cluster with uniform ring heights: The ring heights are tuned to achieve a more homogeneous magnetic energy distribution at around 1.45 GHz. **c,** Cylindrical SRR cluster with variable ring heights: Adjusting each ring's height independently optimizes the magnetic field distribution. **d,** Final toroidal SRR cluster design: This configuration provides the optimal magnetic Purcell enhancement at the desired operating frequency.

SII. Supplementary Discussion

A. Statistical Evaluation of the Mode Uniformity in the Resonator Bore Region

To evaluate whether the calculated mode volume is affected by localized field peaks or numerical artifacts, we performed a statistical analysis of the simulated magnetic-energy density within the bore region of the clustered SRR. The mode volume V_{m} was evaluated only within this region, since the Purcell enhancement depends on the overlap between the resonator's magnetic field and the emitters' dipoles, while magnetic energy outside the gain medium does not contribute to the emission process.

The simulated magnetic energy distribution exhibits moderate uniformity, with a coefficient of variation (CV) of 0.45 and a median-to-mean ratio of 0.94. The ratio of maximum to median magnetic-energy density is 2.40, showing that the field maximum is modestly higher than the typical value and not dominated by a single hotspot. Furthermore, the minimal sub-volumes enclosing 50% and 90% of the magnetic energy are $V_{50} = 0.010$ cm^3 and $V_{90} = 0.025$ cm^3, corresponding to 31% and 78% of the bore volume ($V_{\mathrm{bore}} = 0.032$ cm^3), respectively. These results demonstrate that the magnetic energy is broadly distributed across the emitter region.

The simulation represents an idealized lower bound assuming perfect geometry and complete dipole alignment, whereas the experimental value includes realistic effects such as structural deformation, partial filling, and non-uniform emitter orientation. The resulting threefold difference between the simulated (0.025 cm^3) and experimental (0.072 cm^3) mode volumes is therefore reasonable and consistent with realistic experimental conditions. Combined with the statistical metrics presented above, these results confirm that the mode volume estimation used in this work is physically meaningful and representative of the actual electromagnetic environment experienced by the emitters.

B. Thermal Stability for Continuous-Wave Maser Operation

While the present work focuses on pulsed operation, the excellent thermal properties of the SRR cluster make it particularly promising for stable continuous-wave (CW) applications. The observed MHz-scale linewidths in our pulsed maser experiments arise from the Fourier limit associated with microsecond pulse durations, rather than from intrinsic decoherence or thermal instability. In contrast, CW masers operate in a steady-state regime that have linewidths on the order of tens of kilohertz[1]. Although the absolute linewidths are therefore not directly comparable, the measurements presented here demonstrate exceptional pulse-to-pulse spectral stability of the SRR-based maser over prolonged operation times.

To evaluate the potential for continuous-wave (CW) operation, we analyzed the thermal per-

formance of the silver toroidal SRR cluster and compared it with that of conventional dielectric resonators. Consider, for instance, a maser employing NV centers in diamond with a sapphire resonator, where continuous laser pumping at 400mW caused a frequency shift of 25MHz due to thermal heating [1]. This corresponds to an estimated temperature rise: $\Delta T_{\mathrm{Al_2O_3}} = 35°\mathrm{C}$.

Assuming that the toroidal SRR cluster has a similar size and is operated under the same conditions as the sapphire resonator, the temperature increase ΔT_{SRR} could be estimated as $\Delta T_{SRR}/\Delta T_{Al_2O_3} \sim \kappa_{Al_2O_3}/\kappa_{Ag} \approx 0.1$, following the Fourier's law, where $\kappa_{Al_2O_3}$ and κ_{Ag} are the thermal conductivities of sapphire and silver, respectively. Given that the volumetric thermal expansion coefficient of silver is approximately 54×10^{-6} K^{-1}, the geometric expansion would be less than 0.02%. Consequently, the frequency variation in the SRR cluster is expected to be negligible compared to that observed in the sapphire resonator.

References

[1] J. D. Breeze, E. Salvadori, J. Sathian, N. M. Alford, and C. W. M. Kay, Continuous-wave room-temperature diamond maser, Nature **555**, 493 (2018).
