# Supplementary material for: All-metallic magnetic Purcell enhancement in a thermally stable room-temperature maser
Source: Nat Commun. 2025 Dec 10;16:11214. doi: 10.1038/s41467-025-66016-z (PMC12715216; doi:10.1038/s41467-025-66016-z)
Supplement: Supplementary file 2 — Description of Additional Supplementary Files [file 41467_2025_66016_MOESM2_ESM.pdf]

Description of Additional Supplementary Files

File Name: Supplementary Movie 1
Description: Heat deposition and dissipation in the $SrTiO_3$ resonator under optical excitation.
